# Effect of *MCT1* A1470T Polymorphism on Lactate and Potassium Concentrations After Caffeine Ingestion During Acute Resistance Exercise

**DOI:** 10.3390/nu16244396

**Published:** 2024-12-21

**Authors:** Mohammad Rahman Rahimi, Hassan Faraji, Seyyed Rasoul Hajipoor, Ildus I. Ahmetov

**Affiliations:** 1 Department of Exercise Physiology, University of Kurdistan, Sanandaj 66177-15175, Iran; 2 Department of Physical Education and Sports Science, Marivan Branch, Islamic Azad University, Marivan 14778-93855, Iran; 3 Laboratory of Genetics of Aging and Longevity, Kazan State Medical University, 420012 Kazan, Russia; 4 Research Institute for Sport and Exercise Sciences, Liverpool John Moores University, Liverpool L3 5AF, UK

**Keywords:** DNA, genotype, nutrigenetics, monocarboxylate transporter 1, sports, recovery, resistance exercise, caffeine, lactate

## Abstract

Background: The monocarboxylate transporter 1 (MCT1) plays a crucial role in regulating lactate and pyruvate transport across cell membranes, which is essential for energy metabolism during exercise. The *MCT1* A1470T (rs1049434) polymorphism has been suggested to influence lactate transport, with the T (major) allele associated with greater transport efficiency. This study aimed to investigate the effect of the *MCT1* polymorphism on lactate and potassium (K^+^) concentrations in response to resistance exercise (RE) following caffeine (CAF) ingestion. Methods: Thirty resistance-trained athletes were randomly selected to participate in a randomized, double-blind, placebo-controlled crossover study. Participants consumed either CAF (6 mg/kg of body weight) or a placebo (PL; 6 mg of maltodextrin per kg of body weight) one hour before performing RE. Serum lactate and potassium concentrations were measured before exercise (Pre), immediately after (Post), and 15 min post-exercise (15 min Post). The RE protocol consisted of three sets to failure at 85% of 1RM for each exercise, with 2 min rest intervals between sets. Results: The findings indicate that under caffeine consumption, individuals carrying the A (minor) allele had significantly higher blood lactate levels before (*p* = 0.037) and immediately after (*p* = 0.0001) resistance exercise compared to those with the TT genotype. Additionally, caffeine consumption moderated the increase in plasma potassium levels in TT genotype carriers, while A allele carriers exhibited elevated potassium levels 15 min post-exercise, regardless of caffeine or placebo intake (*p* < 0.05). Conclusions: Our findings suggest that the *MCT1* A1470T polymorphism may influence lactate metabolism and clearance under caffeine consumption, potentially impacting exercise performance and recovery.

## 1. Introduction

Physiological, psychological, socio-cultural, and genetic factors can all potentially affect athletic performance. Additionally, talent is learned and earned through extended and intense practice, as captured by the phrase, “No pain, no gain” [1]. Over the last decade, an increasing body of evidence has demonstrated that genetic variations can influence athletic performance [2,3]. Athletes require a combination of training, nutrition, and genetics to reach peak performance. Training involves the physical preparation and skill development necessary for their sport. Nutrition provides the fuel and building blocks needed for energy production, muscle repair, and overall health. Genetics plays a role in determining an athlete’s physical capabilities and potential for improvement. Research also shows that athletes respond differently to various nutrients and supplements [4,5,6,7]. This variability in response has led to the concept of personalized sports nutrition, where dietary plans are tailored to an individual’s unique needs and goals [8].

Previous research has shown that genetic variants can influence exercise performance [9,10], oxidative stress during intense exercise [11], hormonal response to exercise [5,6], and exercise performance relative to supplement use [4,7]. Other studies highlight the anti-inflammatory effects of caffeine [12] and the impact of genetic variation on responses to micronutrients (vitamins and minerals) [4] and macronutrients [13].

During exercise, an increase in plasma lactate release is accompanied by increased lactate elimination, and the ratio of lactate removal to plasma concentration—lactate clearance—increases from rest to submaximal exercise [14,15,16]. At higher workloads, lactate clearance may level off or even decrease, likely due to saturation at elimination sites [16]. The main tissues responsible for lactate clearance are the liver, heart, and skeletal muscle.

Understanding and optimizing lactate metabolism during intense exercise is crucial for athletes and individuals engaged in high-intensity activities to improve performance, delay fatigue, and enhance overall endurance. Lactate, a byproduct of anaerobic metabolism, accumulates in muscles during intense exercise. Contrary to previous beliefs that lactate causes muscle fatigue and soreness, it is now understood as a fuel source for the muscles, heart, and liver. The lactate shuttle hypothesis proposes that lactate is not merely a waste product but plays a key role in coordinating energy metabolism between tissues. During intense exercise, lactate is shuttled by monocarboxylate transporters (MCTs), which include 14 isoforms. The MCT1 protein is a sarcolemmal lactate/proton cotransporter primarily present in type I oxidative muscle fibers, responsible for transporting lactate and pyruvate across cell membranes—a process critical for cellular energy metabolism.

The *MCT1* A1470T (rs1049434) polymorphism represents a change in the DNA sequence at position 1470, where the nucleotide thymine (T; major allele) is replaced by adenine (A, minor allele). This genetic variation might influence lactate clearance and muscle recovery after intense exercise by potentially altering the efficiency of lactate transport across cell membranes. If the polymorphism leads to decreased transport activity, it could slow lactate clearance from muscle cells, potentially delaying muscle recovery. Conversely, if it enhances transport activity, it could facilitate faster lactate clearance, possibly improving recovery times. A previous study demonstrated that carriers of the minor A allele (i.e., AT + AA genotypes) show higher blood lactate accumulation than those with the AA genotype during high-intensity circuit training (80% of 15 RM) [14], modified Bruce protocol exhaustion tests [15], 30 s Wingate anaerobic tests [16,17], ten maximal-effort 10 s sprints on cycle ergometers [17], and power-matched, one-legged cycling exercises to exhaustion [18]. Additionally, the *MCT1* AA genotype has been associated with elite sprint performance [19], short-distance swimming [20], fat-free mass in well-trained young soccer players [21], and resistance exercise (RE) performance [9]. These effects may be due to changes in lactate transport efficiency across cell membranes.

In addition to the impact of genetic variations on blood lactate levels and exercise performance, previous research indicates that caffeine intake can enhance both resistance exercise (RE) [10,22,23] and endurance performance [24,25], with some of these benefits possibly due to caffeine’s influence on lactate production during exercise.

During muscle contraction, the depolarization of muscle cells leads to the release of potassium (K^+^) into the extracellular fluid, which can then diffuse into blood plasma [26,27]. Regarding caffeine’s effects at the tissue level, the increased secretion of epinephrine and caffeine metabolites like paraxanthine may enhance Na-K pump activity in skeletal muscle, potentially reducing plasma potassium levels (hypokalemia) [28]. In a study, one hour after ingesting 9 mg/kg of caffeine, eight subjects cycled at 78% of their peak VO_2_ until exhaustion, which significantly reduced the exercise-induced increase in plasma [K^+^] [29]. However, caffeine’s effects on lactate production and blood potassium levels can vary depending on individual caffeine sensitivity, dosage, fitness level, and type of exercise.

In summary, understanding and optimizing lactate metabolism during intense exercise is vital for athletes to improve performance, delay fatigue, and enhance endurance. Individuals with the *MCT1* A allele may show differences in lactate clearance rates, muscle fatigue, and overall exercise capacity compared to those with the common TT genotype.

We have therefore tested the hypothesis that the *MCT1* polymorphism can influence the response to caffeine intake during RE. This study aimed to investigate the effect of the *MCT1* polymorphism on lactate and potassium (K^+^) concentrations in response to RE following caffeine (CAF) ingestion.

## 2. Materials and Methods

### 2.1. Ethics Statement

The study was approved by the Ethics Committee in Biomedical Research at the University of Kurdistan (IR.UOK.REC.1399.006; approval date: 6 June 2020). Written informed consent was obtained from each participant. The study complied with the guidelines set out in the Declaration of Helsinki and ethical standards in sport and exercise science research.

### 2.2. Participants

The study included 30 resistance-trained (RT) men with an average age of 21.72 ± 4.06 years, height of 179.31 ± 5.08 cm, and weight of 77.31 ± 14.70 kg. The body composition and anthropometric characteristics of study participants, grouped by different *MCT1* genotypes, are presented in Table S1 (with no significant differences between them). These participants were randomly selected to participate in the research. In addition to having at least one year of resistance training experience, 12 of the recruited subjects were wrestlers with two years of experience, training three times per week. Eight participants were physical education students with three years of training experience, six were judo athletes with 18 months of training three times per week, and four were swimmers with two years of training, also training three times per week. All participants were light caffeine consumers. Eligibility for the study required that participants had not taken anti-inflammatory drugs, supplements, or performance-enhancing substances in the preceding three months. The exclusion criteria for this study included smoking, the use of caffeine-containing medications within the two weeks prior to the study, heavy caffeine consumption (defined as ≥70 mg per day), and the use of caffeine supplements. Initially, the athletes attended a coordination session with the researchers, where the objectives and procedures of the study were explained in detail. After being briefed on the protocol, participants completed a consent form and a health questionnaire. They were also instructed to abstain from caffeinated substances for 24 h before the resistance exercise session.

### 2.3. Experimental Design

**Figure 1:**
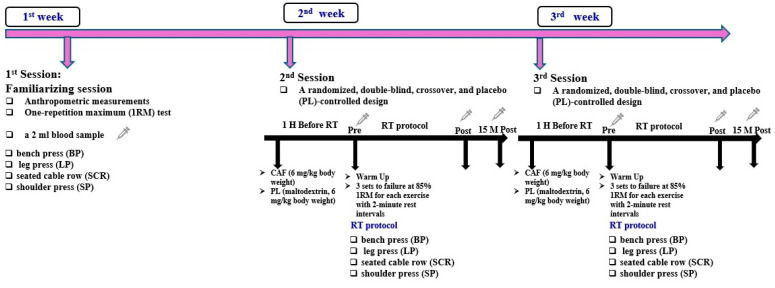
Study design.

This study investigated the effect of acute caffeine supplementation on lactate and potassium (K^+^) concentrations before (Pre), immediately after (Post), and 15 min after (15 min Post) resistance exercise in participants with different *MCT1* genotypes. Three experimental trials were conducted, each separated by one week. During the first session, participants familiarized themselves with the study protocol, underwent body composition analysis using a body composition analyzer, and determined their maximum strength through the one-repetition maximum (1RM) method for bench press (BP), leg press (LP), seated cable row (SCR), and shoulder press (SP) exercises [6,9,30]. For the trials, participants ingested a gelatin capsule containing either caffeine (CAF; 6 mg/kg body weight) or a placebo (PL; maltodextrin, 6 mg/kg body weight) with 250 mL of water one hour before exercise, timed to align with peak blood caffeine levels (Figure 1). Trials took place in the afternoon to control for circadian variation, as in our previous study [6].

The warm-up included 5 min of low-intensity jogging, static stretching for the upper and lower body, and joint-range-of-motion exercises for the chest, shoulders, and back. This was followed by five repetitions at 50% of 1RM for each exercise, including bench press (BP), leg press (LP), seated cable row (SCR), and shoulder press (SP) [6]. The resistance training regimen consisted of three sets to failure at 85% of 1RM per exercise, with 2 min rest intervals. This protocol was followed under both caffeine (CAF) and placebo (PL) conditions. Performance was monitored by the same supervisor, who provided verbal encouragement, and repetitions per set were recorded to assess performance.

### 2.4. Biochemical Testing

To measure blood lactate and K^+^ levels using a photometric method (Parsazmun kit, Tehran, Iran) and an enzymatic method (Biorexfars kit, Province, Iran), 2 mL of blood was collected from the anterior elbow vein before, immediately after, and 15 min after resistance exercise. Blood samples were taken from participants who had ingested either caffeine or a placebo one hour prior to exercise.

### 2.5. Genetic Analysis

Genomic DNA was extracted from peripheral blood using the TIANamp Genomic DNA Kit (Tiangen Biotech, Beijing, China) following the manufacturer’s instructions (cat. no. DP304) to determine the athletes’ genotype. The *MCT1* A1470T polymorphism was identified via polymerase chain reaction (PCR) and restriction enzyme digestion. The PCR utilized primers (forward 5′-AGCAAACGAGCAGAAAAAGG-3′ and reverse 5′-CTGGGTCATGAACTGCTCAA-3′), producing a 187 bp fragment (Figure S1). PCR products were digested with the BccI restriction enzyme (New England Biolabs, Ipswich, MA, USA) for 8 h at 37 °C (11), separated by 6% polyacrylamide gel electrophoresis, stained with ethidium bromide, and visualized under UV light (Figure S2).

### 2.6. Statistical Analysis

A total sample size of 28 and an actual power of 97% were determined based on an expected effect size of 0.4 (medium effect size) for the gene–treatment interaction (2 × 2), three measurements (repeated three times) had a significance level of 0.05. Repeated measures analysis of variance (ANOVA) and Tukey’s post hoc test were used to assess the effects of genotype (TT and TA + AA) and supplement (caffeine vs. placebo) on blood lactate and K+ levels. Physical characteristics and body composition (weight, fat-free mass, fat mass, % body fat, BMI, and BMR) were compared using the independent samples test. A significance level of *p* < 0.05 was used for all tests, and statistical analyses were conducted using GraphPad Instat software version 9 (GraphPad Software, Inc., San Diego, CA, USA).

## 3. Results

### 3.1. Effects of Caffeine Consumption on Plasma Lactate Concentration

**Figure 2:**
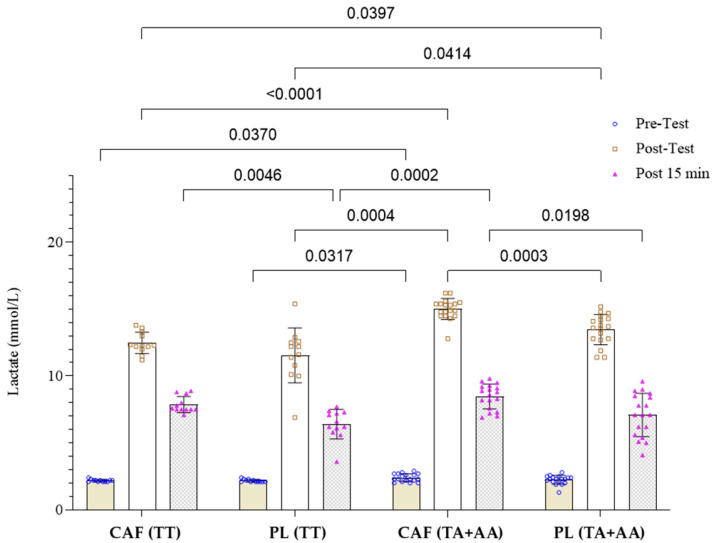
Lactate response to resistance exercise under caffeine (CAF) and placebo (PL) conditions in TT (n = 12) and TA + AA (n = 18) genotypes of the *MCT1* gene.

The two-way repeated measures ANOVA for blood lactate concentration indicated a significant effect of time (F(1.86,104.2) = 1882, *p* < 0.0001), a significant effect of intervention × genotype (F(3,56) = 24.37, *p* < 0.0001), and a significant interaction between time × intervention × genotype (F(6,112) = 9.38, *p* < 0.0001). Of the 30 athletes, 12 were carriers of the TT genotype, 13 were TA heterozygotes, and five were AA homozygotes, with a distribution that met Hardy–Weinberg expectations (*p* > 0.05). Tukey’s post hoc test revealed that under caffeine consumption, blood lactate levels in A allele carriers were significantly higher before (*p* = 0.037) and immediately after (*p* = 0.0001) resistance exercise compared to those with the TT genotype (Figure 2). Additionally, under caffeine conditions compared to the placebo, blood lactate levels in individuals with the A allele were significantly higher immediately after and 15 min after resistance exercise than in both the TT genotype group (*p* = 0.0004 and *p* = 0.0002) and the TA + AA genotypes group under the placebo (*p* = 0.0003 and *p* = 0.0198). Furthermore, lactate levels in A allele carriers were higher immediately after resistance exercise under placebo conditions compared to TT genotype carriers under both caffeine (*p* = 0.039) and placebo (*p* = 0.041) conditions. Intra-group comparisons showed that blood lactate levels increased from pre-test to immediately after and 15 min after resistance exercise in both genotypes, under both caffeine and placebo conditions (*p* < 0.05).

### 3.2. Effects of Caffeine Consumption on Plasma Potassium Concentration

**Figure 3:**
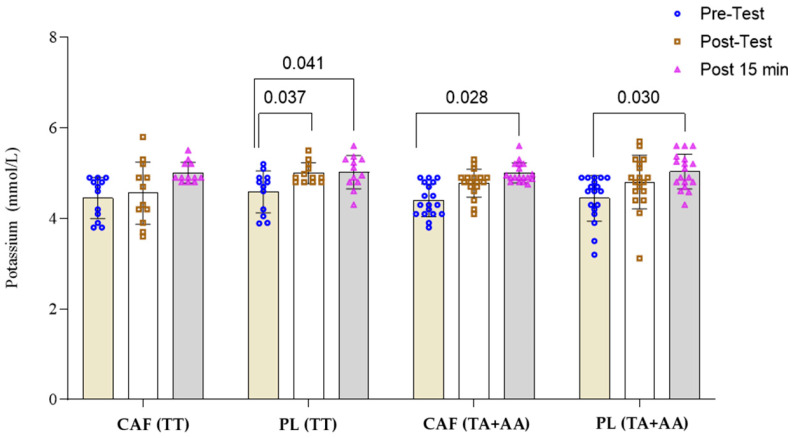
Potassium response to resistance exercise under caffeine (CAF) and placebo (PL) conditions in TT (n = 12) and TA + AA (n = 18) genotypes of the *MCT1* gene.

The two-way repeated measures ANOVA for plasma potassium concentration indicated a significant effect of time (F(1.916,107.3) = 23.11, *p* = 0.0001), while the effect of intervention × genotype (F(3,56) = 1.59, *p* = 0.201) and the interaction between time × intervention × genotype (F(6,112) = 0.608, *p* = 0.716) were not significant. Tukey’s post hoc test for intra-group comparisons showed that potassium levels in individuals with the A allele were significantly higher 15 min after resistance exercise under both caffeine and placebo conditions compared to pre-exercise (*p* < 0.05) (Figure 3). In individuals with the TT genotype, under placebo conditions, potassium levels significantly increased immediately after and 15 min after resistance exercise compared to pre-exercise levels (*p* < 0.05).

## 4. Discussion

The *MCT1* A1470T polymorphism, also known as rs1049434, is a genetic variant in the *MCT1* gene that plays a crucial role in lactate transport across muscle tissues. This polymorphism results in the substitution of glutamic acid with aspartic acid at codon 490, which influences lactate transport efficiency. The variant has garnered attention due to its potential impact on athletic performance, particularly in distinguishing between different types of athletes. Studies indicate that individuals carrying the A allele (A1470T) exhibit a significantly reduced rate of lactate transfer, estimated to be 35–65% lower than non-carriers with the TT genotype. This reduced transport capacity can lead to higher blood lactate levels during and after exercise, potentially affecting overall athletic performance [31]. Interestingly, elevated lactate levels may also stimulate anabolic signaling pathways associated with muscle hypertrophy, such as mTOR and IGF-1. This suggests that while high lactate levels may indicate impaired clearance, they may also play a role in muscle growth and adaptation, particularly in strength athletes [17].

To our knowledge, the effect of caffeine supplementation in relation to the *MCT1* A1470T polymorphism on lactate levels after resistance exercise has not been previously investigated. Our study results demonstrated that, in general, the increase in lactate levels in the caffeine group was higher than in the placebo group. Individuals with the TT genotype had lower lactate concentrations compared to those with the TA + AA genotypes. Additionally, potassium levels increased significantly after resistance exercise in individuals with the TT genotype. However, in the caffeine group, no significant change in potassium levels was observed after exercise in TT genotype individuals. In contrast, in the TA + AA genotype, potassium levels were higher 15 min after resistance exercise than before, in both caffeine and placebo conditions. Furthermore, no significant association was found between the *MCT1* A1470T polymorphism and participants’ body composition.

These findings suggest that the *MCT1* A1470T polymorphism may influence lactate and potassium dynamics during and after resistance exercise, particularly when caffeine is ingested. The differential responses observed between the TT and TA + AA genotypes highlight the potential for personalized nutrition and exercise strategies based on an individual’s genetic profile.

Individuals with the TT genotype tend to exhibit better lactate clearance, which is associated with improved performance in endurance sports. For instance, endurance athletes often show a higher frequency of the TT genotype compared to non-athletes, suggesting a genetic advantage in lactate management during prolonged physical activity [32]. However, another study reported that in men, the TT group had higher lactate values than the AA group in all measurements after circuit training, with no differences observed among female genotypes [33]. Carriers of the A allele have been reported to have higher capillary lactate accumulation during intense circuit training compared to their T allele counterparts [14]. Recently, a study on Japanese wrestlers demonstrated that athletes with the TT genotype had lower blood lactate concentrations than athletes with the A allele after a 30 s Wingate physical activity. This study concluded that the TT genotype was associated with lactate clearance and better recovery after high-intensity efforts [32].

In line with these findings, our study showed that subjects with TA and AA genotypes of the *MCT1* A1470T polymorphism had higher blood lactate levels immediately after resistance exercise in both caffeine and placebo conditions compared to those with the TT genotype. The higher lactate levels in TA and AA genotypes immediately after exercise suggest that these individuals may experience greater lactate accumulation during resistance exercise. Studies indicate that people with the A allele (TA and AA genotypes) have a lower lactate-carrying capacity, resulting in increased blood lactate concentrations during and after intense exercise due to impaired clearance from blood flow to muscle cells and other tissues [17]. Additionally, individuals with the *MCT1* A allele do not perform well in transferring lactate to less active muscle cells for oxidation, thus increasing their blood lactate concentration [34].

Caffeine acts as an adenosine antagonist, which can enhance glycolytic pathways and increase lactate production even at rest. By blocking adenosine receptors, caffeine may stimulate glycolysis, leading to greater lactate production in resting muscles [35]. Caffeine may enhance glycolytic pathways, leading to increased lactate production during intense exercise due to heightened muscle activation and energy demands, resulting in more lactate as a byproduct of anaerobic metabolism [35]. Additionally, caffeine may influence lactate clearance mechanisms by disrupting lactate transfer from muscles to the bloodstream or from the bloodstream to the liver for gluconeogenesis, contributing to elevated blood lactate post-exercise.

Under caffeine conditions, the higher blood lactate levels before resistance exercise in TA + AA genotypes compared to TT genotypes indicate that individuals with TA + AA genotypes have a lower lactate-carrying capacity, resulting in higher resting blood lactate concentrations due to the disruption of blood flow to tissues such as the liver and heart, which utilize lactate [36]. Additionally, individuals with the TT genotype in the placebo group experienced the smallest increase in lactate after resistance exercise. Previous research suggests that caffeine can elevate blood lactate levels post-exercise, although results vary, potentially due to factors such as caffeine sensitivity, polymorphisms affecting caffeine metabolism, and training status [5,6,9,10].

One study found no significant difference in blood lactate levels between different caffeine doses and a placebo after mid-endurance running, though lactate levels post-exercise were notably higher than pre-exercise levels. This suggests that caffeine may not effectively reduce lactate accumulation during activities of this type [35]. Another study investigated caffeine’s effects on blood lactate during incremental exercise and found that caffeine consumption does not delay the onset of lactate accumulation. In fact, blood lactate levels were significantly elevated during submaximal exercise with caffeine, indicating that caffeine may accelerate lactate production or inhibit its clearance from the bloodstream [37]. In cycling tests, researchers examined caffeine’s effects on blood glucose and lactate levels after 1 km and 20 km of cycling. Results showed significant differences in lactate levels pre- and post-exercise, but caffeine did not consistently affect these levels at various doses, highlighting the complexity of caffeine’s impact on lactate dynamics [38]. In contrast, a study involving a Wingate test found that caffeine did not significantly increase blood lactate compared to placebo conditions; however, lactate levels tended to be higher in the placebo condition, suggesting caffeine may reduce lactate accumulation during high-intensity efforts [39].

Regarding plasma potassium levels, previous studies have indicated that acute physical activity prompts K+ release from contracting muscles, proportional to exercise intensity. This release reduces intracellular K+ in active muscles and raises plasma K+ levels during exercise [40]. Reduced intracellular potassium during exercise can impact muscle performance and physiological responses. A decrease in intracellular potassium may impair muscle contraction, leading to increased fatigue and reduced strength, which can hinder performance and increase injury risk due to muscle weakness [41]. Low intracellular potassium can also disrupt neuronal function, potentially causing muscle cramps. Conversely, a substantial rise in plasma potassium (hyperkalemia) can disrupt heart electrical activity, leading to arrhythmia or impaired neuromuscular transmission, resulting in muscle weakness. Post-exercise, the body generally works to restore potassium balance, but if plasma potassium remains elevated, cell recovery can be compromised. A less efficient sodium–potassium pump can prolong the normalization of potassium levels, potentially leading to a transient hypokalemic state after acute exercise [40].

In our study, under placebo conditions, potassium levels in the TT genotype placebo group increased significantly immediately and 15 min after resistance exercise. However, in the caffeine group with the TT genotype, no significant increase in potassium levels was observed post-exercise. This effect was mitigated by caffeine consumption, suggesting that caffeine moderates plasma potassium increases in individuals with the TT genotype. Caffeine appears to exert this effect by inhibiting phosphodiesterase, raising cyclic adenosine monophosphate (cAMP) levels, which redistributes potassium and promotes renal excretion through ATP activation, facilitating the movement of extracellular potassium into cells [42]. The diuretic effect of caffeine enhances renal potassium excretion and increases the glomerular filtration rate, leading to caffeine-induced diuresis and potassium excretion [42,43].

Additionally, caffeine may stimulate the beta-adrenergic system, increasing renin secretion and activating the renin–angiotensin–aldosterone system, leading to increased potassium loss [41]. The T allele may affect potassium transport and regulation, such as those encoding the Na^+^-K^+^ ATPase pump. Higher pump activity increases potassium influx, lowering extracellular potassium levels while raising intracellular concentrations [44]. Furthermore, individuals with the TT genotype may differ in the efficiency of these processes compared to other genotypes, potentially affecting plasma potassium levels during physical activity. However, these hypotheses remain inconclusive, and further research is needed to substantiate them.

Another key finding of this study was that potassium levels in individuals with the TA + AA genotypes were higher 15 min post-resistance exercise compared to pre-exercise levels in both caffeine and placebo conditions. This suggests that individuals carrying the A allele do not exhibit differences in plasma potassium response to acute resistance exercise, regardless of caffeine intake. In the placebo condition, potassium levels in TT genotype individuals increased significantly immediately and 15 min post-exercise, indicating that TT carriers in the *MCT1* A1470T polymorphism experience a greater increase in plasma potassium levels following acute exercise. This effect is compounded by their tendency for lower blood lactate levels, which may influence potassium dynamics. The physiological response to exercise in TT genotype individuals may involve a more pronounced increase in plasma potassium due to their unique metabolic profile. Studies have shown that TT genotype carriers have lower post-exercise lactate concentrations compared to A allele carriers, suggesting a more efficient recovery process potentially related to potassium regulation. The interaction between lactate clearance and potassium homeostasis is crucial, as both ions play roles in muscle metabolism and recovery.

In this study, no significant differences in height, weight, lean body mass, body fat mass, fat percentage, BMI, or basal metabolic rate (BMR) were observed between TT genotype carriers and TA + AA genotype carriers, likely due to the sample size being insufficient for this type of association analysis. Previous studies have reported that the A allele is associated with speed/power performance [19]. Some research suggests a relationship between the A allele and hypertrophy and fat mass. For instance, soccer players with the AA genotype or the A allele in the *MCT1* A1470T polymorphism have shown significantly higher lean mass percentages compared to those with the TT genotype [34]. However, other studies have not found significant differences in physical characteristics such as height, weight, body mass, or metabolic rate across alleles [9]. The relationship between genotype and phenotype is complex and influenced by environmental factors, exercise regimens, and interactions with other genetic variations. For example, while the AA genotype has been linked to higher lean mass in some studies, this association may not apply universally across athletic populations or performance metrics. The physiological mechanisms underlying these associations remain unclear, complicating interpretation across studies [4].

Our study had limitations that should be considered in interpreting the results. Genetic association studies benefit from large sample sizes, which was not feasible in this study. Further research on genotype–phenotype associations in diverse racial and ethnic populations could provide broader insights, especially concerning muscle hypertrophy and body composition changes in response to exercise. Additionally, we did not measure intracellular lactate levels or factors affecting the lactate pump in cell membranes. Examining these factors in future studies could yield a better understanding of the underlying mechanisms.

## 5. Conclusions

In conclusion, our findings suggest that caffeine increases lactate production during resistance exercise compared to a placebo. Individuals with the TT genotype of the *MCT1* A1470T polymorphism showed lower lactate concentrations than those with the TA + AA genotypes, suggesting a favorable effect of the TT genotype on lactate clearance in response to caffeine intake and resistance exercise. Potassium levels increased significantly after resistance training in TT genotype carriers, but this effect was moderated by caffeine consumption. In contrast, potassium levels were elevated 15 min post-exercise in TA + AA genotype carriers, irrespective of caffeine or placebo intake. Future research should further investigate the mechanisms behind lactate and potassium differences in TT and TA + AA genotypes, focusing on lactate transport efficiency and its effects on muscle metabolism. Examining long-term caffeine effects on lactate and potassium dynamics in individuals with different *MCT1* A1470T genotypes may reveal insights for personalized nutrition and exercise strategies. Additionally, exploring interactions between *MCT1* A1470T and other genes affecting lactate metabolism could support targeted interventions for improved exercise performance and recovery. Including diverse populations, like endurance athletes, could help generalize these findings and identify genetic markers for exercise response and recovery.

## Data Availability

The data that support the findings of this study are available from the corresponding author upon reasonable request due to privacy of athletes.

## Supplementary Materials

The following supporting information can be downloaded at: https://www.mdpi.com/article/10.3390/nu16244396/s1, Figure S1: PCR products obtained from the amplification of the *MCT1* gene A1470T polymorphism. Figure S2: Products of the enzymatic digestion of amplified fragments of the MCT1 gene A1470T polymorphism. Table S1: Body composition and anthropometric characteristics of study participants.
