# Supplementary material for: Effect of MCT1 A1470T Polymorphism on Lactate and Potassium Concentrations After Caffeine Ingestion During Acute Resistance Exercise
Source: Nutrients. 2024 Dec 21;16(24):4396. doi: 10.3390/nu16244396 (PMC11676378; doi:10.3390/nu16244396)
Supplement: Supplementary file 1 [file nutrients-16-04396-s001.zip › nutrients-3327233-supplementary.pdf]

**Supplementary file**

**Table S1.** Body composition and anthropometric characteristics of study participants

| | Genotype | Mean | T, df, *p* value |
|---|---|---|---|
| Height (cm) | TT | 180.36±2.54 | 0.86, 28, 0.39 |
| | TA+AA | 178.66±6.13 | |
| Weight (kg) | TT | 75.07±6.57 | -0.71, 28, 0.47 |
| | TA+AA | 79.19±18.19 | |
| Soft Lean Mass (kg) | TT | 60.87±4.39 | -0.87, 28, 0.39 |
| | TA+AA | 63.84±10.69 | |
| Body Fat Mass (kg) | TT | 10.83±4.30 | -0.39, 28, 0.69 |
| | TA+AA | 11.85±7.78 | |
| Percent Body Fat | TT | 14.20±4.93 | 0.12, 28, 0.90 |
| | TA+AA | 13.95±5.35 | |
| BMI ($kg/m^2$) | TT | 23.08±2.03 | -1.06, 28, 0.29 |
| | TA+AA | 24.73±4.89 | |
| BMR | TT | 2039.40±118.95 | -0.73, 28, 0.47 |
| | TA+AA | 2104.23±276.75 | |

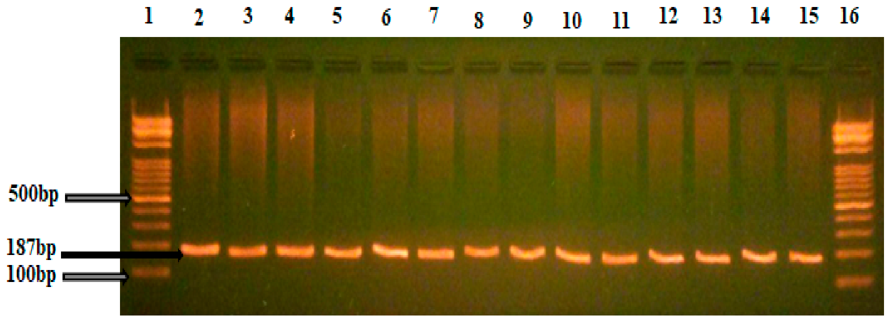


**Figure S1**. PCR products obtained from the amplification of the *MCT1* gene A1470T polymorphism. Wells 2 to 15 contain PCR products from this amplification, with an amplified fragment length of 187 bp. Wells 1 and 16 contain molecular markers of 100 base pairs.

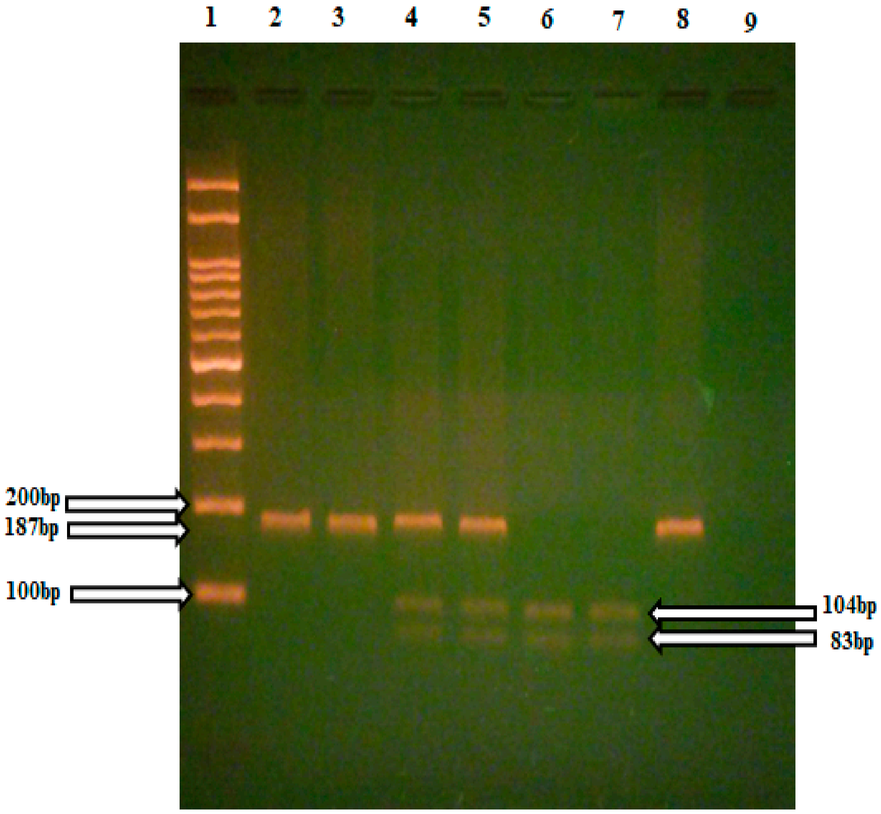


**Figure S2.** Products of enzymatic digestion of amplified fragments of the *MCT1* gene A1470T polymorphism. Wells 2 and 3: TT genotype; Wells 4 and 5: AT genotype; Wells 6 and 7: AA genotype; Well 8: undigested PCR product; Well 9: negative control; Well 1: 100 bp molecular marker.
